# Genomic insights into bamboo witches’ broom disease: pathogenicity and phytohormone biosynthesis in *Aciculosporium take*

**DOI:** 10.3389/fmicb.2024.1432979

**Published:** 2024-11-08

**Authors:** Yu Gu, Haoyue Yu, Jiayan Kuang, Xiaoping Ma, Muhammad Salman Tahir, Sainan He, Yingchong Liao

**Affiliations:** ^1^College of Life Sciences, Sichuan Agricultural University, Yaan, China; ^2^College of Agronomy, Sichuan Agricultural University, Chengdu, China; ^3^Key Laboratory of Animal Disease and Human Health of Sichuan Province, College of Veterinary Medicine, Sichuan Agricultural University, Chengdu, China

**Keywords:** bamboo witches’ broom disease, *Aciculosporium take* Miyake, chromosome level genome assembly, pathogenicity, comparative genomic, phytohormone biosynthesis

## Abstract

Bamboo witches’ broom disease (WBD), caused by *Aciculosporium take* Miyake, devastates bamboo forests. Understanding the genome and pathogenic factors of pathogen is crucial for disease control. We employed single-molecule real-time sequencing, Illumina paired-end sequencing, and chromatin interaction mapping techniques to assemble the genome of *A. take* CCTCC-M2023413, analyze pathogenicity- and phytohormone-biosynthesis-related genes, and compare it to 12 other WBD pathogens. The genome of *A. take* is 59.24 Mb in size, with 54.32% repeats, 7 chromosomes, 7,105 protein-coding genes, 84 ribosomal RNAs, and 115 transfer RNAs. Predictive analysis of pathogenicity genes found 237 carbohydrate-active enzymes, 1,069 membrane transport proteins, 1,040 pathogen-host interaction genes, 315 virulence factors, and 70 effectors. Most of pathogenicity genes overlapped with repeat-rich regions. Additionally, 172 genes were linked to auxin biosynthesis, 53 to brassinosteroid biosynthesis, and 2 to *cis*-zeatin biosynthesis. Comparative genomic analysis identified 77 core orthogroups shared by 13 WBD pathogens, played roles in metabolites, genetic information processing, pathogenesis, *cis*-zeatin biosynthesis, lifespan, and quorum sensing. The *miaA* gene, crucial for *cis*-zeatin biosynthesis, is structurally conserved and sequence-diverse among 13 WBD pathogens, with upregulated expression during bamboo WBD pathogenesis. This highlights that *cis*-zeatin is significant contributor to host pathogenesis, and miaA is a new potential target for controlling WBD. This study provides important insights on preventing and controlling bamboo WBD.

## 1 Introduction

China is recognized as the primary center of origin for bamboo, encompassing over 500 species across 39 genera. The bamboo forest area is 75,627 km^2^, constituting 3.31% of the nation’s total forest area. Notably, moso bamboo forests represent 69.78% of the total bamboo forest area (Feng and Li, 2023). The genera *Phyllostachys*, typified by *Phyllostachys edulis*, along with *Arundinaria* and *Yushania*, which serve as the primary diet for giant pandas, are particularly susceptible to bamboo witches’ broom disease (WBD) (Yang and Wu, 2011; Park, 2016).

*Aciculosporium take* Miyake (Ascomycota, Clavicipitaceae; Anamorph: Albomyces take Miyake) is the causative agent of bamboo WBD (Tsuda, 1997). In China, WBD is recognized as a significant threat to bamboo forests (Ann et al., 2006), leading to weakened growth, decreased shoot production, and potential plant mortality (Tanaka, 2009). The decline of bamboo forests poses significant threats to the food supply of giant pandas, endangers biodiversity, and results in huge economic losses for forest farmers. A high-quality genome assembly, annotation, and analysis may play a significant role in addressing the issue of *A. take*’s pathogenesis.

The genomic information of *A. take* MAFF-241224 (GCA_000222935.2) in the NCBI database was stored in 2011, and was assembled to the contig level using the Roche 454 sequencing system, resulting in a genome coverage of 18.5×, which was insufficient for a comprehensive genetic characterization of this pathogenic fungus. After 10 years of technological advancements, the sequencing technology has been improved. The groundbreaking advancements in whole-genome sequencing, initiated by the successful sequencing of *Saccharomyces cerevisiae*’s genome in 1996 (Goffeau et al., 1996), have revolutionized our understanding of fungal genomics. The utilization of third-generation sequencing technology from the PacBio platform enables the generation of complete chromosome sequences, albeit with a higher error rate than second-generation sequencing (Liu et al., 2012). This can be mitigated by integrating high-fidelity short reads from platforms like Illumina with long reads (Rhoads and Au, 2015). More and more fungi have established telomere-to-telomere genomes by these sequencing technology, such as *Stagonospora tainanensis* (Xu et al., 2022), *Sporisorium panici-leucophaei* (Crestana et al., 2021), and *Fusarium oxysporum* (Fokkens et al., 2020). The primary methodologies and goals of genomics research in plant pathogens encompass a comprehensive examination of the genomic characteristics of pathogenic microorganisms using bioinformatics analysis, identification of gene functions, elucidation of pathogenic mechanisms, and genetic analysis. Comparative genomics has become a valuable tool for investigating the evolution of pathogenesis and identifying novel virulence determinants, as evidenced in certain plant pathogenic fungal genera such as *Fusarium* (Brown et al., 2012) and *Botrytis* (Valero-Jimenez et al., 2019). By integrating second-generation and third-generation sequencing technologies with comprehensive data analysis, high-quality genomes can be obtained to elucidate the pathogenesis of *A. take.*

The pathogenic mechanisms of plant pathogenic fungi are intricate and multifaceted (Hématy et al., 2009). This interaction encompasses numerous secreted fungal molecules, including plant cell wall-degrading enzymes (PCWDEs) (Kubicek et al., 2014), nutrient-acquiring transport proteins, effector proteins, and other small molecules. While plant pathogenic fungi have the capacity to secrete a significant quantity of proteins, only a fraction is deemed pathogenic (Wang et al., 2019). Certain effectors are activated within the plant, modulating the metabolic pathways of plant cells, inhibiting the plant’s defense response, and directly impacting the invasion and manifestation of diseases induced by the pathogen (Gassmann and Bhattacharjee, 2012). *A. take* may also infect hosts through these mechanisms, necessitating a theoretically elucidation of its potential pathogenesis at the genomic level.

The molecular mechanism underlying the development of bamboo WBD, induced by *A. take*, encompasses various factors including the suppression of bud apical dominance, excessive germination and growth of lateral buds, and deterioration of leaves and stems, potentially modulated by hormonal regulation. Prior analyses utilizing High Performance Liquid Chromatography (HPLC) have demonstrated that pathogen invasion can elevate Cytokinin (CK) levels in plants, thereby disrupting normal physiological and metabolic processes, ultimately manifesting as symptoms of WBD (Kesumawati et al., 2006). Tanaka et al. (2003a) observed that branches affected by WBD displayed elevated levels of auxin and CK in comparison to healthy branches. They proposed that the presence of *A. take* promotes the development of bud primordia through the production of auxin within the buds, while CK aids in the aggregation of axillary buds, leading to reduced leaf size and the manifestation of WBD symptoms. Additionally, changes in hormone concentrations have been identified in cocoa plants afflicted by WBD induced by *Moniliophthora perniciosa* (Teixeira et al., 2014). Analysis of the cocoa transcriptome during the onset of WBD indicated an enhancement of phytohormone signal transduction pathways in the host plant, particularly in auxin, gibberellin (GA), CK, and ethylene (ETH). Genes associated with GA biosynthesis and signal perception showed increased expression, along with a significant up-regulation of auxin response genes. While extensive research has been conducted on WBD in cocoa and paulownia trees, the pathogenesis of WBD in bamboo remains rare and is only hypothesized to be linked to alterations in phytohormone levels. However, a comprehensive understanding is hindered by the lack of a complete genome sequence.

This study utilized single-molecule real-time (SMRT) sequencing, Illumina paired-end sequencing and High-throughput chromosome conformation capture (Hi-C) technologies to annotate high-quality genome assemblies at the chromosome level of *A. take*. These annotations were combined with extensive databases of pathogenic factor analyses to identify *A. take* phytohormone synthesis genes and investigate the pathogenic mechanism of *A. take*. Furthermore, a comparative genomic analysis of 13 different species causing WBD revealed shared genes and highlighted the central role of CK in the pathogenesis of WBD, providing valuable insights into the determinants of WBD symptoms.

## 2 Materials and methods

### 2.1 Fungal isolation and identification

The conidiostromatas and stromatas on WBD bamboo shoots of *Phyllostachys sulphurea* were collected in Zhangjiashan, Ya'an City, Sichuan Province, China. Partial samples stored at −80°C for RT-qPCR assays, and another portion was used for pathogen isolation.

Three isolates of *A. take* were isolated from the conidiostromata (Xiansheng Geng et al., 2020). The isolates were cultivated on sabouraud dextrose agar (SDA) at 25°C for 10 days. Following incubation, the fungal cultures were carefully scraped from the surface of the SDA using a sterile scalpel. The harvested mycelia were transferred to sterile microcentrifuge tubes to ensure purity and absence of contaminants. Genomic DNA was subsequently extracted using the CTAB method (Kistler, 2012). Polymerase Chain Reaction (PCR) conditions were optimized with an initial denaturation step at 94°C for 5 min, followed by 35 cycles of denaturation at 94°C for 30 s, annealing at 55–60°C for 30 s, and extension at 72°C for 1 min, with a final extension at 72°C (the time is dependent on fragment length). After the isolates were testified and comparison with known reference sequences using ITS (600 bp region), *β*-tubulin (250 bp region), and EF1-*α* (400 bp region) detection (Rehner and Buckley, 2005) and by pathogenic morphology (include colony morphology, size and shape of conidia), 3 isolated were identified to the same strain. The strain was stored in the China Center for Type Culture Collection under the designation CCTCC No. M 2023413.

### 2.2 PacBio based genome sequencing and assembly

For whole-genome sequencing, the concentration and quality of genomic DNA were assessed using a NanoDrop 2000 spectrophotometer (NanoDrop Technologies, Wilmington, DE, United States), a Qubit 3.0 Fluorometer (Life Technologies, Carlsbad, CA, United States), and 0.8% agarose gel electrophoresis. Eligibility criteria of quality DNA were: (1) OD260/280 ratio between 1.8 and 2.0, OD260/230 ratio between 2.0 and 2.2, no moderately polluted protein; (2) a Qubit concentration > 80 ng/μl and 0.95 < Nc/Qc < 1.5; (3) normal color and no RNA. Qualified DNA samples with 5 μg were fragmented and size-selected using the BluePippin system (Sage Science, MA, United States). Subsequently, the SMRTbell Express Template Prep Kit 2.0 (Pacific Biosciences, CA, United States) was utilized to generate SMRTbell HiFi libraries. The size and quality of the libraries were evaluated using the FEMTO Pulse system and the Qubit dsDNA HS Assay Kit (Life Technologies, Carlsbad, CA, United States). Sequencing primers and Sequel II DNA polymerase were separately annealed and then combined with the final SMRTbell library. Following the completion of library construction, sequencing was conducted on the PacBio Sequel II platform with a loading concentration of 120 pM (Sun et al., 2020). The SMRT Link software (Ardui et al., 2018) was used for preprocessing the raw sequencing data, and the CCS^1^ was applied for HiFi analysis to obtain subread information.

Subreads were assembled using HiFiasm (version 0.16.1) (Cheng et al., 2021), followed by a completeness assessment with the fungi_odb10 gene set,^2^ using BUSCO (v5.2.2) (Simao et al., 2015) and base composition statistics.

### 2.3 Hi-C sequencing and assisted assembly of chromosomes

*Aciculosporium take* conidia sample underwent treatment with 1% formaldehyde (v/v) to stabilize DNA conformation. Subsequently, cross-linked DNA was digested with restriction endonuclease MboI (New England Biolabs Inc., Ipswich, MA) to produce sticky ends. Blunting and repair of DNA ends were carried out, with the introduction of biotin for oligonucleotide end labeling. The resulting DNA fragments were ligated using DNA ligase, followed by proteinase K (Thermo Fisher, Waltham, MA) digestion to reverse DNA cross-linking. The DNA was then purified and randomly fragmented into segments measuring 300–500 bp. Biotin-labeled DNA was isolated using streptavidin-coupled Dynabeads, and a library was constructed for sequencing on the BGI MGISEQ-2000 platform with PE150.

Following quality control procedures, the clean reads were mapped into the raw genome of *A. take* by PacBio sequencing and *de novo* assembly. The Hi-C assembly method leverages chromosome proximity to cluster scaffolds/contigs, generate interaction matrices, and create interaction maps with 3D-DNA software (Dudchenko et al., 2017). JuicierBox (Durand et al., 2016) was utilized for visualization and correction to achieve a chromosome-level genome assembly. Completeness was assessed using the fungi_odb10 gene set with BUSCO (Simao et al., 2015).

### 2.4 Identification of repeat sequence and non-coding RNA

Repeat sequences within the genome were discerned through a dual methodology involving *ab initio* (Price et al., 2005) and a homology-based approach (Jurka et al., 2005) facilitated by RepeatMasker v4.1.2 (Tarailo-Graovac and Chen, 2009) and RepeatProteinMask v4.1.2s (Tarailo-Graovac and Chen, 2009) software. Subsequently, these transposable elements (TE) were classified into distinct subcategories.

The tRNAscan-SE software (Lowe and Eddy, 1997) was employed to detect transfer RNA (tRNA) sequences within the genome based on their structural attributes. Furthermore, BLASTN analysis was utilized to identify ribosomal RNA (rRNA) sequences, and the covariance model of the Rfam^3^ (Griffiths-Jones et al., 2005) was employed to predict microRNA and small nuclear RNA (snRNA) sequences within the genome.

### 2.5 Genome prediction and basic functional annotation

The genetic structure was annotated using a combination of three strategies: (1) *de novo* prediction utilizing Augustus, GlimmHMM and Genescan; (2) transcriptome-based prediction with PASA using RNA-seq data generated from the current study; and (3) homology-based prediction with five phylogenetic species. The integration of gene sets and curation of resulting alignments were performed by MAKER v3.0 and PASA, respectively.

Gene functions were annotated by aligning sequences to numerous databases, such as SwissProt^4^ (O'Donovan et al., 2002), NR^5^ (O'Donovan et al., 2002), GO^6^ (Ashburner et al., 2000), KEGG^7^ (Kanehisa et al., 2012), InterPro^8^ (Blum et al., 2021), TrEMBL^9^ (O'Donovan et al., 2002), and eggNOG v5.0^10^ (Aramaki et al., 2020).

### 2.6 Fungal pathogenicity-related gene annotation

Pathogenicity-related genes in the genome of *A. take* were annotated against a set of databases. The carbohydrate-active enzymes (CAZYs) were annotated using the Carbohydrate-Active Enzymes database (CAZy) (Kubicek et al., 2014). Membrane transport proteins were identified through the Transporter Classification Database (TCDB) (Saier et al., 2021), while pathogen–host-interaction (PHIs) genes were analyzed using the Pathogen–Host Interaction Database (PHI-base) (Urban et al., 2020). Additionally, distinct virulence factor genes were revealed through analysis using the Virulence Factor Database (VFDB) (Chen et al., 2005).

The candidate effectors were identified through a systematic pipeline, wherein proteins possessing signal peptides and less than two transmembrane helices were detected using SignalP v6.0 (Almagro Armenteros et al., 2019) and TMHMM v2.0 (Chen et al., 2003), respectively. Then, proteins with an extracellular location were classified as putatively secreted proteins using ProtComp v9.0 from Softberry. Subsequently, these secreted proteins were further categorized as effectors using EffectorP v3.0 (Sperschneider and Dodds, 2022) and distinguished as either cytoplasmic or apoptotic effectors.

### 2.7 Identification of genes related to plant hormone biosynthesis

The investigation of pivotal gene Pfam identifiers associated with the plant hormone biosynthesis pathway in the KEGG database was pursued, followed by the retrieval of the Hidden Markov Model (HMM) from the Pfam database.^11^ The complete protein sequences of *A. take* were employed to perform the initial domain exploration utilizing the HMM model. Subsequently, a species-specific HMM model was developed via multiple sequence alignment using clusterW2, and the pertinent genes were confirmed through an additional inquiry in the Conserved Domain Database (CDD)^12^ (Marchler-Bauer et al., 2011) and Pfam database (Punta et al., 2012).

### 2.8 Comparative genomic analysis

In order to explore the pathogenic mechanisms of *A. take*, comparative genomic analysis was performed on 13 distinct plant pathogens (Supplementary Table S1), encompassing fungi and phytoplasma, all known to induce WBD in various host plants. The genomic protein sequences of 12 pathogens downloaded from NCBI database^13^. OrthoFinder (Emms and Kelly, 2019) was employed to identify orthologous proteins among the 13 pathogens.

The alignment of single-copy core orthologous proteins was conducted using MAFFT (Katoh and Standley, 2013), followed by the inference of species phylogeny using the maximum likelihood program in IQ-tree (Nguyen et al., 2015). Genome-wide replication events between species were analyzed utilizing the One Step MCScanX module in TBtools v1.1047 software (Wang et al., 2012). Functional annotation of common orthologous proteins of 13 pathogens was performed against the eggNOG (Aramaki et al., 2020) and KEGG database (Kanehisa et al., 2012).

### 2.9 Analysis of *cis*-zeatin synthesis genes in 13 pathogens of WBD

The miaA domain sequences of 13 pathogens were confirmed in the NCBI-CDD. Motifs were identified using the MEME tool^14^ with the default parameter settings of maximum number of motifs = 50 and minimum number of motifs = 10. NCBI-CDD was used to analyze motif function. Multiple sequence alignment results were obtained using the MUSCLE Wrapper module in TBtools v1.1047 software to determine the miaA structural conservation.

### 2.10 Determination of *miaA* expression in *A. take* during the formation of WBD

Based on several years observation, the pathogen maintains in an asexual stage when it is not exposed from the leaf sheath of bamboo buds, during which the bamboo buds experience rapid growth. Conversely, the pathogen transitions to a sexual stage upon exposure from the bamboo buds, characterized by the development of yellow fruiting bodies, at which point the growth of bamboo bud ceases. Asexual and sexual sporophores were aseptically collected from naturally morbidity WBD bamboo buds of *P. sulphurea* in Zhangjiashan, China, and subsequently utilized to assess the expression of *miaA* (*gene-Ata03552*) using the RT-qPCR method.

Total RNA was extracted following the protocols outlined in the Universal RNA Extraction Kit (AG, Hunan, China). RNA samples (1 μg) were treated with 1 U DNase I for 10 min at 37°C to eliminate any genomic DNA (Berbert et al., 2022). Subsequently, reverse transcription was performed using the Evo M-MLV RT Reverse Transcription Kit (AG, Hunan, China). The qPCR reactions were conducted using the SYBR Green Pro Taq HS Premixed qPCR Kit (AG, Hunan, China) according to the manufacturer’s instructions. For quantification, the internal reference gene 18sRNA was utilized (Ben-Daniel et al., 2012). Spores from the strain cultured in SDA at 25°C for 10 days were served as a control to represent non-infection phase. Each treatment was done in triplicate, and three independent biological samples were test for data robustness. Primer sequences are detailed in Supplementary Table S2. The relative expression of *miaA* was calculated using the 2^−∆∆Ct^ formula. Statistical analysis was conducted using ANOVA with significance set at a *p*-value of <0.05.

## 3 Results

### 3.1 The symptoms of the bamboo witches’ broom disease and morphology of *Aciculosporium take*

**Figure 1:**
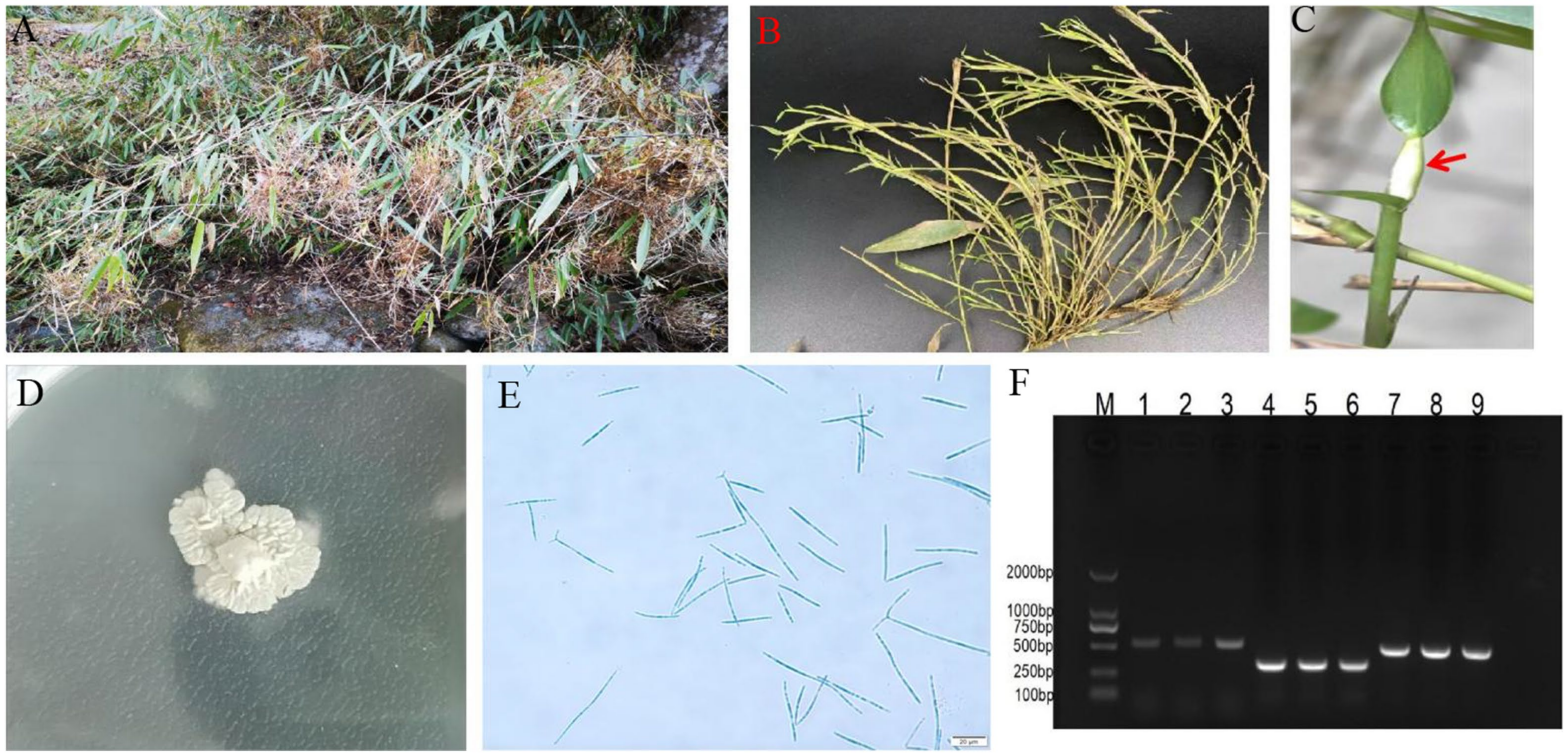
Symptoms of bamboo witches’ broom disease (WBD) and morphology of *Aciculosporium take*. (A) Severe symptoms of bamboo WBD. (B) Axillary buds tuft and wilting in WBD. (C) Sporodochium of *A. take*, indicated by red arrows. (D) Colony (CCTCC-M2023413) on SDA at 25°C for 1 mo. (E) Conidia morphology. Scale = 20 μm. (F) Electrophoresis bands of the internal transcribed spacer1 (ITS1), *β*-tubulin, and elongation factor 1-*α* (EF1-α). M: DL2000 marker; PCR products of the ITS primer (1 ~ 3), β-tubulin primer (4 ~ 6), and EF1-α primer (7 ~ 9).

Common symptoms of the bamboo witches’ broom disease (WBD) in the infected bamboo plants include massive tuft and wilting of axillary buds (Figures 1A,B), accompanied by the formation of sporodochium at the apex of the buds (*A. take* asexual growth stage) (Figure 1C). The strain was identified and named *A. take* CCTCC-M2023413 based on its colony characteristics on SDA, characterized by creamy white colonies with a wrinkled surface (Figure 1D), microscopic conidia morphology (Figure 1E), and sanger sequenced of internal transcribed spacer (ITS) segments, *β*-tubulin, and elongation factor 1-*α* (Figure 1F).

The genomic information of *A. take* MAFF-241224 (GCA_000222935.2) in the NCBI database was stored in 2011, and was assembled to the contig level using the Roche 454 sequencing system, resulting in a genome coverage of 18.5X, which was insufficient for a comprehensive genetic characterization of this pathogenic fungus.

### 3.2 Genome assembly and quality assessment

PacBio sequencing generated 8,111,417 subreads (106.38 Gb) using one Sequel II SMRT cell. Following HiFi statistics subreads, 6.43 Gb bases were acquired, with a genome size of 59,238,630 bp (59.24 Mb), achieving a coverage of 109×, the maximum length of 50,189 bp, and N50 of 13,679 bp (Supplementary Figure S1A). After HiFiasm initial assembly, 21 contigs were comprised with N50 of 7,246,485 bp. The assessment of the assembly indicated a completeness of 99.7%, demonstrating excellent assembly results.

After filtering low-quality sequences from the MGISEQ-2000 platform, 51,588,867 bp of clean reads were obtained, with 96.82% of reads mapping to the preliminary PacBio assembled genome. Following Hi-C assisted assembly and correction, 10 contigs were obtained, ultimately constructing 7 chromosomes (Supplementary Figure S1B), with 4 contigs not assembled into chromosomes. Six out of 7 chromosomes have telomeric repeats (AACCCT)_n_ at least one end, with a contig anchoring rate of 99.32%. The longest chromosome was 12,863,373 bp and the shortest was 5,993,614 bp, and N50 was 8.82 Mb, with a chromosome GC content of 40.28% (Table 1). Finally, the completeness of the *A. take* genome assembled with the fungi_odb10 was calculated to be 99.7%. These findings collectively indicate the assembly of a highly complete and accurate *A. take* genome.

**Table 1:** Summary of genome assembly annotation features of *Aciculosporium take*.

Features	*Aciculosporium take*
Chromosome features	
No. of chromosomes	7
No. of contigs	10
Size of genome (Mb)	59.24
GC content of genome (%)	40.28
N50 chromosome length (Mb)	8.82
Maximum chromosome length (bp)	12,863,373
Minimum chromosome length (bp)	5,993,614
Gene structure information	
Protein-coding genes in the chromosomes	7,105
Avg gene length (bp)	2,543
Avg CDS length (bp)	1,327
Avg exon number	3
Avg exon length (bp)	612
Avg intron length (bp)	169
Non-coding RNA information	
rRNAs (copy)	84
tRNAs (copy)	115
snRNA (copy)	16
CD-box	10
HACA-box	1
Splicing	5

### 3.3 Repeat and non-coding RNA analysis

In *A. take*, tRNA had a maximum copy number of 115, rRNA had a copy number of 84. Additionally, small quantities of snRNAs (16) were identified, including CD-box (10), HACA-box (1), and splicing genes (5) (Table 1).

**Figure 2:**
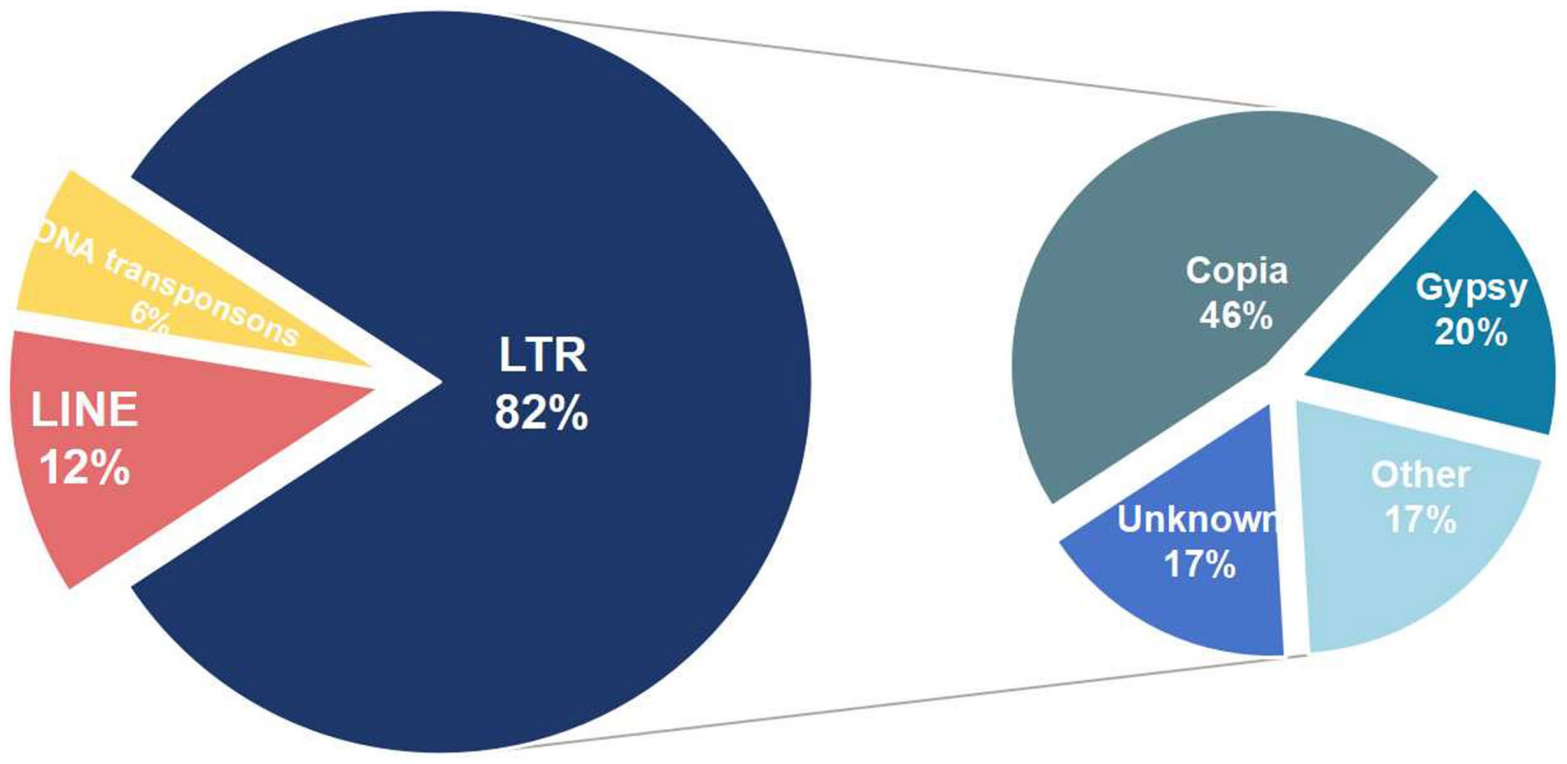
Types of transposable element in *Aciculosporium take*. The first circle on the left represents the primary TE components, while the second circle on the right illustrates the various types of LTRs.

After combining results from both methods and eliminating redundancies, 54.32% of the genome were repeat sequences with 3.7% (2,189,208 bp) tandem repeat and 52.35% transposable element (TE) (Table 2). The genome consists of long terminal repeat (LTR) sequences (35.55%), and long interspersed nuclear elements (LINE) sequences (5.58%), with a small amount of DNA transposons (0.4%). However, short interspersed nuclear elements (SINE) were not present. Subsequent examination of the more prevalent LTRs (Figure 2), reveals that the Copia retrotransposons constitute the largest individual TE family, comprising 46% of the LTR, with Ty3-retrotransposon following at 20%.

**Table 2:** Identification of the transposable element sequences of *Aciculosporium take*.

Features	Combined TE Length (bp)	Combined TE percentage in the genome
DNA transposons	237,270	0.4
LTR	21,052,095	35.55
LINE	3,306,197	5.58
SINE	930	0
Other	273	0
Unknown	8,211,034	13.87
Total TE	30,994,361	52.35

### 3.4 Gene structure analysis and basic functional annotation

The gene structures analysis pipeline of the MAKER and PASA identified a total of 7,105 protein-coding genes, with an average gene length of 2,543 bp and an average exon count of 3 (Table 1). Annotation of the assembled genes was conducted using six databases, resulting in 6,160 genes being successfully annotated while 945 genes remained unannotated. The NR database had the highest number of annotations at 6,156 (86.64%), followed by the TrEMBL database with 6,154 (86.62%) and the KEGG database with 6,103 (85.9%). A total of 4,496 genes were annotated across all six databases, while 1,680 genes were annotated in five databases. Additionally, 5 genes were exclusively annotated by the NR database and 2 genes were exclusively annotated by the InterPro database (Supplementary Figure S2).

### 3.5 Annotation of pathogenicity-related genes

**Figure 3:**
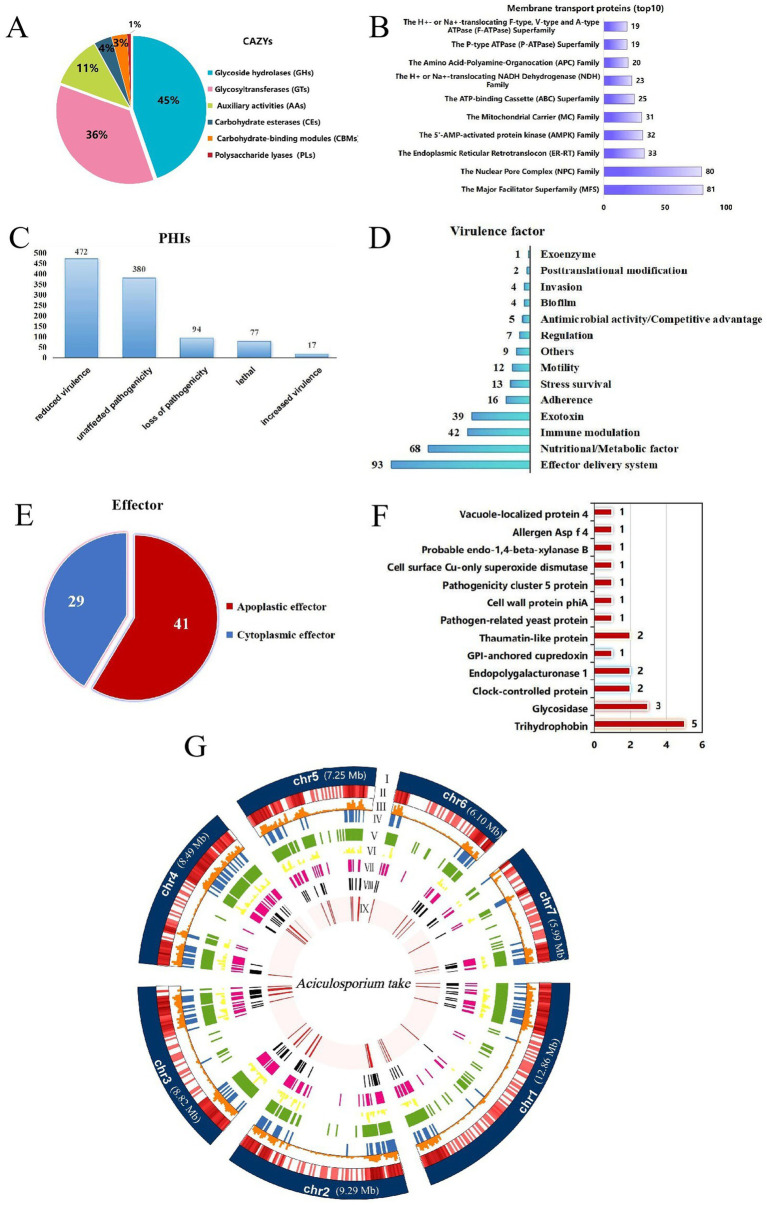
Summary of pathogenicity-related genes annotations. (A) Carbohydrate-active enzymes (CAZys). (B) Membrane transport proteins (top 10). (C) Pathogen-host Interaction related genes (PHIs). (D) Virulence factors. (E) Effectors. (F) The apoplastic effector function annotation results. The numbers on the bar chart represent the quantity of genes annotated to the respective function. (G) Circle diagram showing the basic features of the *Aciculosporium take* genome. (I) Seven chromosomes. Chromosome length expressed in Mb, (II) Gene density in each chromosome. The darker the color, the higher the gene density, (III) Repeat sequences density in each chromosome, (IV) Cazys-related genes, (V) membrane transport proteins. (VI) PHI-related genes, (VII) virulence factor genes, (VII) secreted proteins, (IX) effectors.

In order to enhance comprehension of the pathogenic mechanism of *A. take*, a substantial quantity of pathogenicity-associated proteins was identified. Annotation of the *A. take* genome by the CAZy revealed the presence of 237 genes related to carbohydrate-active enzymes, encompassing 106 (45%) glycoside hydrolases (GHs), 85 (36%) glycosyltransferases (GTs), 27 (11%) auxiliary activity enzymes (AAs), 9 (4%) carbohydrate esterases (CEs), 8 (3%) carbohydrate-binding modules (CBMs), and 2 (1%) polysaccharide lyases (PLs) (Figure 3A).

A total of 1,069 membrane transport proteins were identified, with the major facilitator superfamily (MFS) comprising 81 members, the nuclear pore complex family with 80 members, and the endoplasmic reticulum-retention and retrieval family with 33 members (Figure 3B).

Analysis of the genome utilizing PHI-base revealed 1,040 PHI-related genes, including 17 associated with enhanced virulence, 472 with decreased virulence, 77 with lethality, 94 with loss of pathogenicity, and 380 with no effect on pathogenicity (Figure 3C).

Additionally, analysis utilizing the VFDB database identified 315 unique virulence factor genes with diverse functional roles (Figure 3D). Effector delivery systems, nutrient/metabolism factors, immune regulation, and extracellular toxin-related genes were the top four categories ranked by number.

Effector proteins of *A. take* were analyzed by initially identifying 639 proteins with signal peptides using SignalP 6.0. Subsequently, 168 proteins with fewer than two transmembrane structural domains and extracellular localization were identified as secreted proteins (Figure 3G). From these secreted proteins, a total of 70 effectors were predicted using EffectorP 3.0, consisting of 41 apoptotic effectors and 29 cytoplasmic effectors (Figure 3E). The apoptotic effectors were functionally annotated to obtain functional proteins related to trihydrophobin, various hydrolases, clock-controlled protein, and pathogenesis (Figure 3F).

To understand gene distribution characteristics, the assembled genomic chromosomes, repeat sequences, and predicted locations of pathogenesis-related coding proteins are visualized on a circle map (Figure 3G). Chromosome mapping shows that genes and repeat sequences are concentrated at both ends of the chromosome, with a higher gene density and the presence of higher transposable elements. Genes linked to pathogenicity also tend to be located at the chromosome ends, overlapping with transposable element-rich regions.

### 3.6 Genes involved in phytohormone biosynthesis pathways

**Figure 4:**
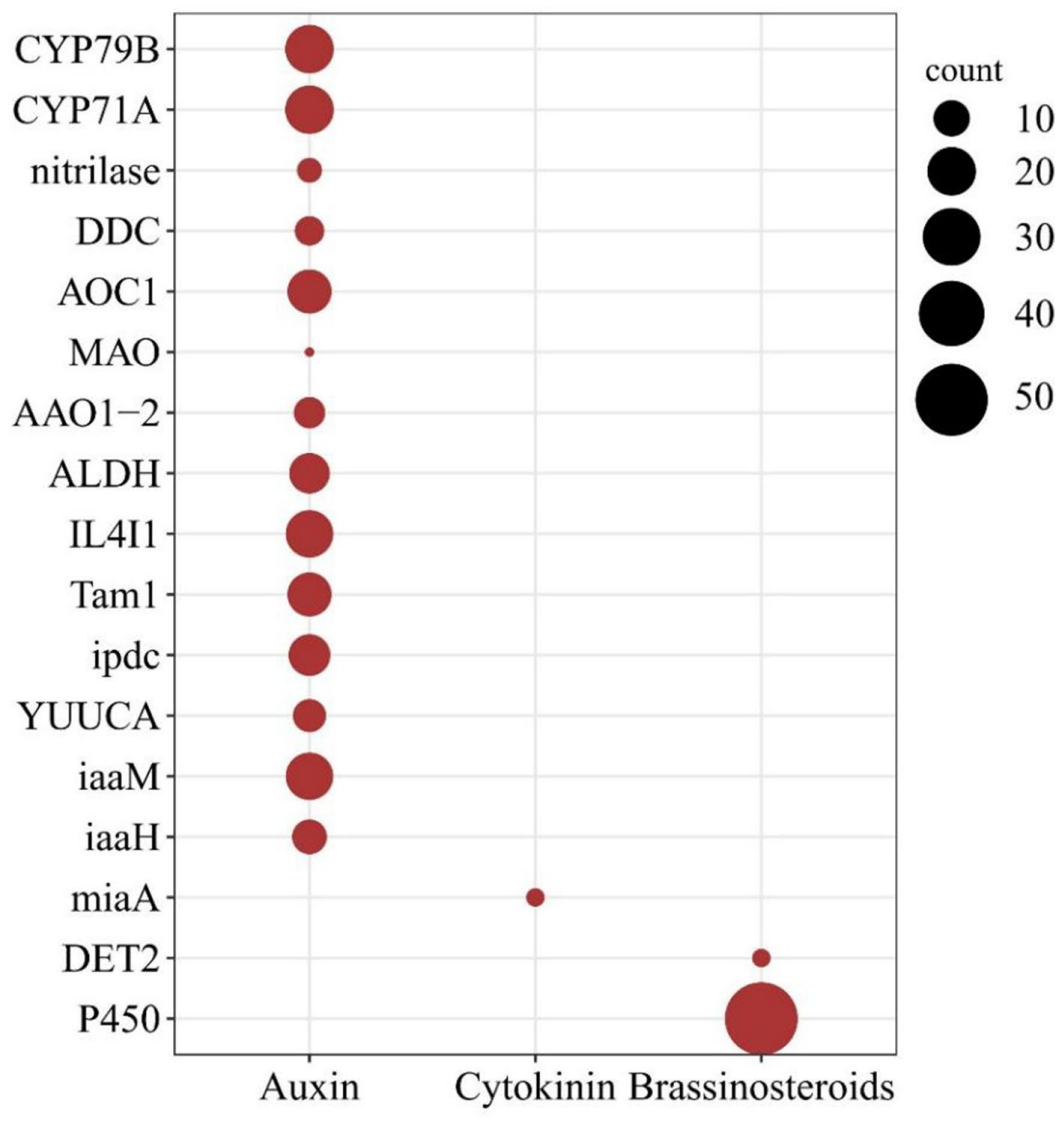
Phytohormone biosynthesis genes in the *Aciculosporium take* genome. The larger dark red solid circles indicating a greater number of genes. The horizontal axis denotes the type of hormone, while the vertical axis represents the gene families of hormone biosynthesis pathway.

The rapid growth and morphological changes observed in WBD plants may be linked to alterations in phytohormone metabolism. Transcriptome analysis of healthy and WBD bamboos indicated an imbalance in phytohormone metabolism during pathogenesis (PRJNA980656). The investigation of hormone biosynthesis genes in the *A. take* genome confirmed the presence of gene families primarily associated with the regulation of growth-related hormones such as auxin, CK, and Brassinosteroids (BR). A total of 17 gene families comprising 227 genes were found to be involved in the synthesis of these phytohormones in *A. take*. Specifically, 172 genes from 14 gene families were identified in the auxin biosynthesis pathway, 53 genes were implicated in BR biosynthesis, and only 2 miaA genes were associated with CK biosynthesis (Figure 4).

### 3.7 Comparative genomic analysis of the pathogens that cause WBD

**Figure 5:**
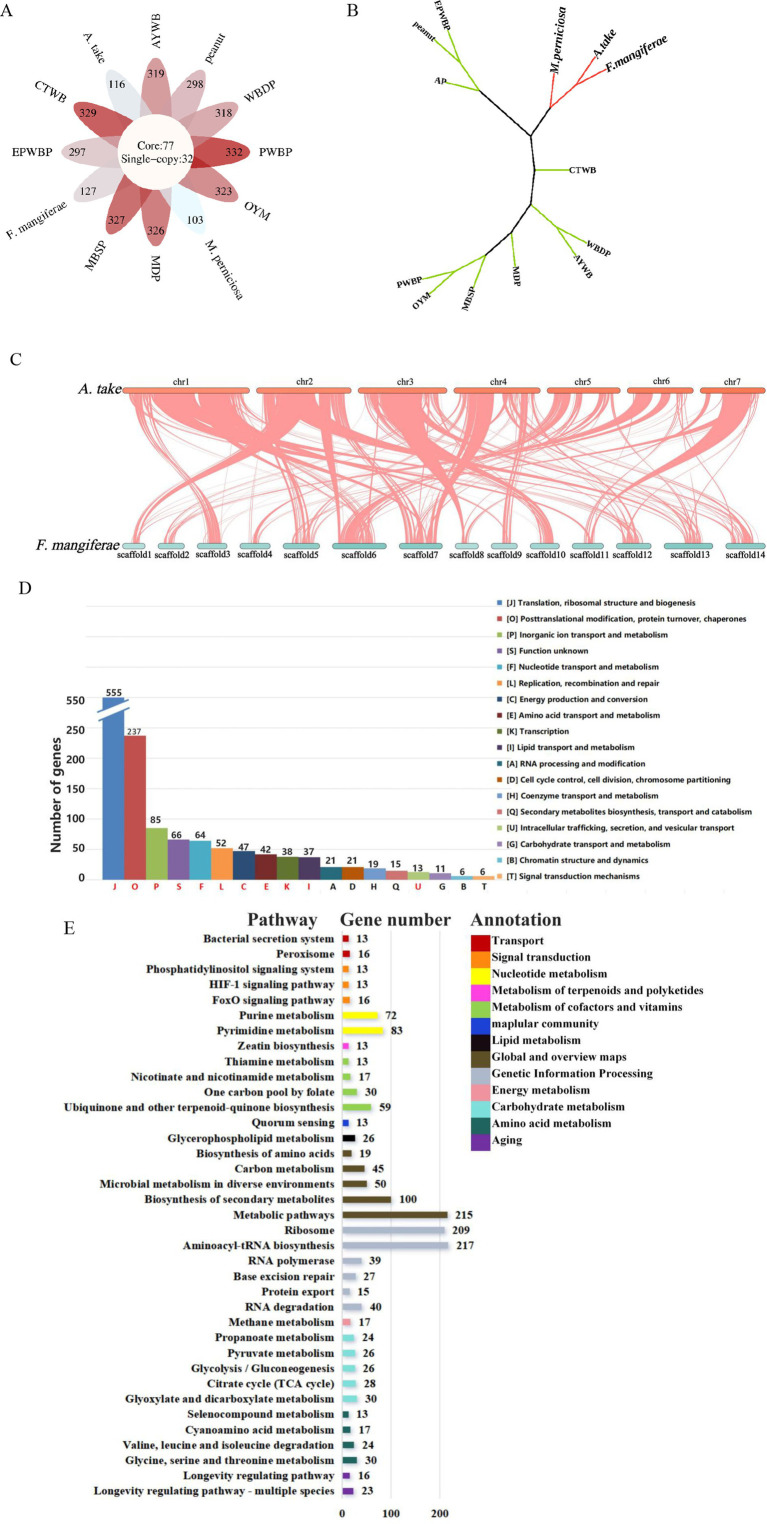
Comparative genomic analysis of plant pathogens associated with witches’ broom disease (WBD). (A) Summary of orthogroups across 13 species. The core of the diagram depicts the quantity of single-copy orthogroups, while the periphery indicates the number of species-specific genes for each respective species. (B) A phylogenetic tree analysis between *Aciculosporium take* and 12 other WBD pathogens based on the single-copy core orthogroups. EPWBP, *Echinacea purpurea* witches’ broom phytoplasma; peanut, Peanut witches’ broom phytoplasma; AP, Apple proliferation phytoplasma; *M. perniciosa*, *Moniliophthora perniciosa* cocoa witches’ broom; *F. mangiferae*, *Fusarium mangiferae* mango deformity disease; *A. take*, *Aciculosporium take*; CTWB, Chinatree witches’ broom phytoplasma; MDP, Mulberry dwarf phytoplasma; MBSP, Maize bushy stunt phytoplasma; PWBP, Paulownia witches’ broom phytoplasma; OYM, onion yellows phytoplasma; AYWB, Aster yellows witches’ broom phytoplasma; and WBDP, wheat blue dwarf phytoplasma. (C) Collinearity between *A. take* and *F. mangiferae*. Chr1–chr7 represent the chromosomes of *A. take*, whereas scaffold1-scaffold14 correspond to the chromosomes of *F. mangiferae*. (D) The 77 core orthogroups eggNOG annotation summary results. Different letters represent different functional categories, red letters are shared functional pathways annotated to 13 species. Vertical coordinates indicate the number of genes. (E) The co-shared KEGG pathway by 77 core orthogroups. The numbers on the histogram indicate the number of genes enriched into this pathway, and the different colors in the legend represent different types of functions.

OrthoFinder assigned 25,263 genes from the 13 species to 6,589 orthogroups, with 50% of the genes falling into orthogroups containing two or more genes (G50 = 2); the largest orthogroup consisted of 5,047 genes (O50 = 5,047). In total, 77 core orthogroups encompassed all species, 32 of which were entirely comprised of single-copy genes (Figure 5A). Single-copy orthogroups were selected for constructing phylogenetic trees, revealing that *A. take* is most closely related to *F. mangiferae*, followed by *M. perniciosa* (Figure 5B). High collinearity was evident between the genomes of *A. take* and *F. mangiferae*, as indicated by their shared ownership of 5,447 orthogroups (Figure 5C). The relationship between the chromosomes of these two organisms was elucidated through the analysis of collinearity blocks.

The 77 core orthogroups containing 1,310 genes were analyzed for eggNOG and KEGG enrichment (Figures 5D,E; Supplementary Table S3). The top 5 functional categories (J, O, P, S, F) identified were associated with growth and development, protein synthesis metabolism, and genetic information processing. The common functional categories of 13 WBD pathogens were enriched in J, O, P, S, F, L, C, E, K, I, and U, with the U related to intracellular transport, secretion, and vesicle trafficking processes. Proteins found within this category of secretions may be implicated in the common pathogenic mechanisms underlying the occurrence of WBD. Furthermore, analysis of gene annotations using the KEGG for the 77 core orthogroups revealed a total of 37 pathways shared by 13 pathogens. Of these pathways, 22 are involved in diverse metabolic processes such as amino acid, carbohydrate, energy, nucleotide, cofactors, vitamins, and lipid metabolism, which are essential for biological growth and development. Thirteen pathogens were found to be enriched in the Zeatin Biosynthesis Pathway, suggesting that CK may play a significant role in the development of WBD. Additionally, 39 genes were enriched in the Longevity Regulating Pathway and FoxO Signaling Pathway, which are known to regulate the aging process in organisms. Thirteen genes were also found to be enriched in the HIF-1 Signaling Pathway, which plays a role in regulating oxygen homeostasis and the tricarboxylic acid (TCA) cycle. Pathways such as Bacterial Secretion system, Peroxisome, and Quorum Sensing were identified as being related to pathogenic processes. These findings contribute to the identification of common pathogenic genes involved in WBD and essential genes necessary for sustained growth.

### 3.8 Identification and analysis of *cis*-zeatin biosynthesis genes in 13 agents of WBD

Analysis of 77 core orthologous shared by 13 WBD pathogens revealed the significance of a key protein, miaA, in the development of WBD. MiaA plays a crucial role in catalyzing the conversion of adenosine 37 in tRNA to 6-isopentenyladenosine, serving as the sole catalytic enzyme in the dimethylallyl diphosphate synthesis *cis*-zeatin pathway. The presence of miaA indicates the capability of these pathogens to synthesize *cis*-zeatin. Investigating the structural characteristics of miaA in different WBD pathogens can help identify targets for controlling bamboo WBD.

**Figure 6:**
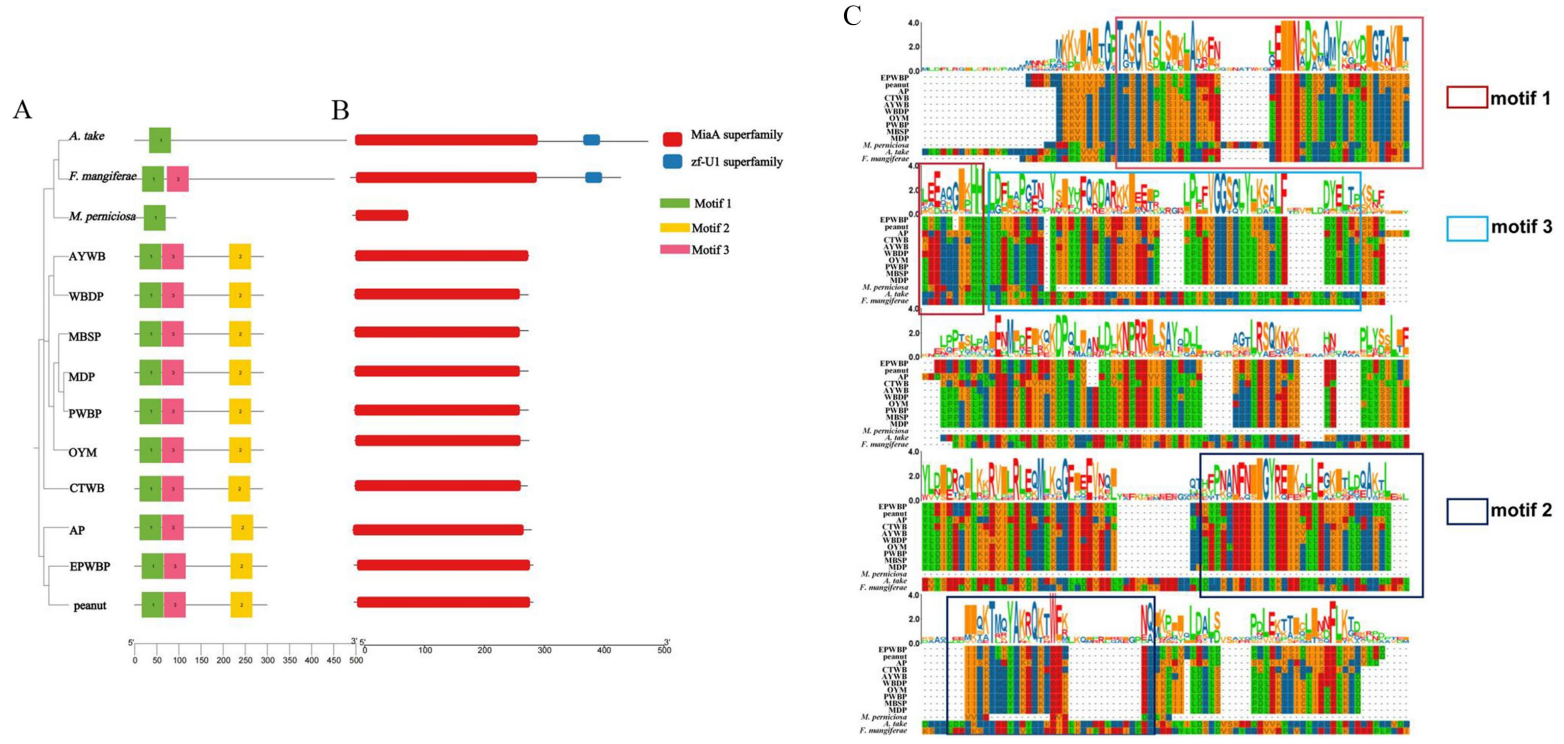
Phylogenetic tree, conserved motifs and domain of miaA between *Aciculosporium take* and 12 WBD pathogens. (A) Phylogenetic tree and motif analysis of miaA. The length of the solid line corresponds to the protein sequence length, while colored boxes denote distinct motifs. The figure on the right provides a detailed amino acid modeling of the motif. (B) Domain pattern analysis of miaA. (C) Protein sequence alignment of miaA between *A. take* and 12 WBD pathogens. Different colored boxes represent different motifs.

A phylogenetic analysis was conducted utilizing the miaA protein sequences of 13 pathogens to assess their evolutionary relationships (Figure 6A). The characterization of these miaA proteins revealed that the genetic proximity between *A. take* and *F. mangiferae* was the closest, while *M. perniciosa* clustered within a larger branch. Additionally, it was observed that the miaA protein sequences of filamentous fungi diverged into distinct branches from those of phytoplasmas.

The conservation of protein regions is fundamental to their functionality, and further investigation into the structural features of miaA includes the study of motif and domain patterns (Figures 6B,C). Motif 1 is conserved in miaA across 13 species, with members of the same taxonomic group exhibiting similar motif patterns. All 10 phytoplasmas examined share motifs 1, 2, and 3, with motif 2 displaying unique characteristics. Among the three fungi, *F. mangiferae* possesses an additional motif 3. Furthermore, an analysis of the structural domains within miaA proteins revealed the presence of a miaA superfamily in all species. Notably, the zf-U1 superfamily zinc-binding domain was identified in both *A. take* and *F. mangiferae*. Overall, the miaA sequences among various species exhibit a notable level of conservation, as evidenced by the outcomes of multiple sequence comparisons of miaA across diverse species.

### 3.9 Expression profiles of *miaA* in *A. take* during bamboo WBD pathogenesis

**Figure 7:**
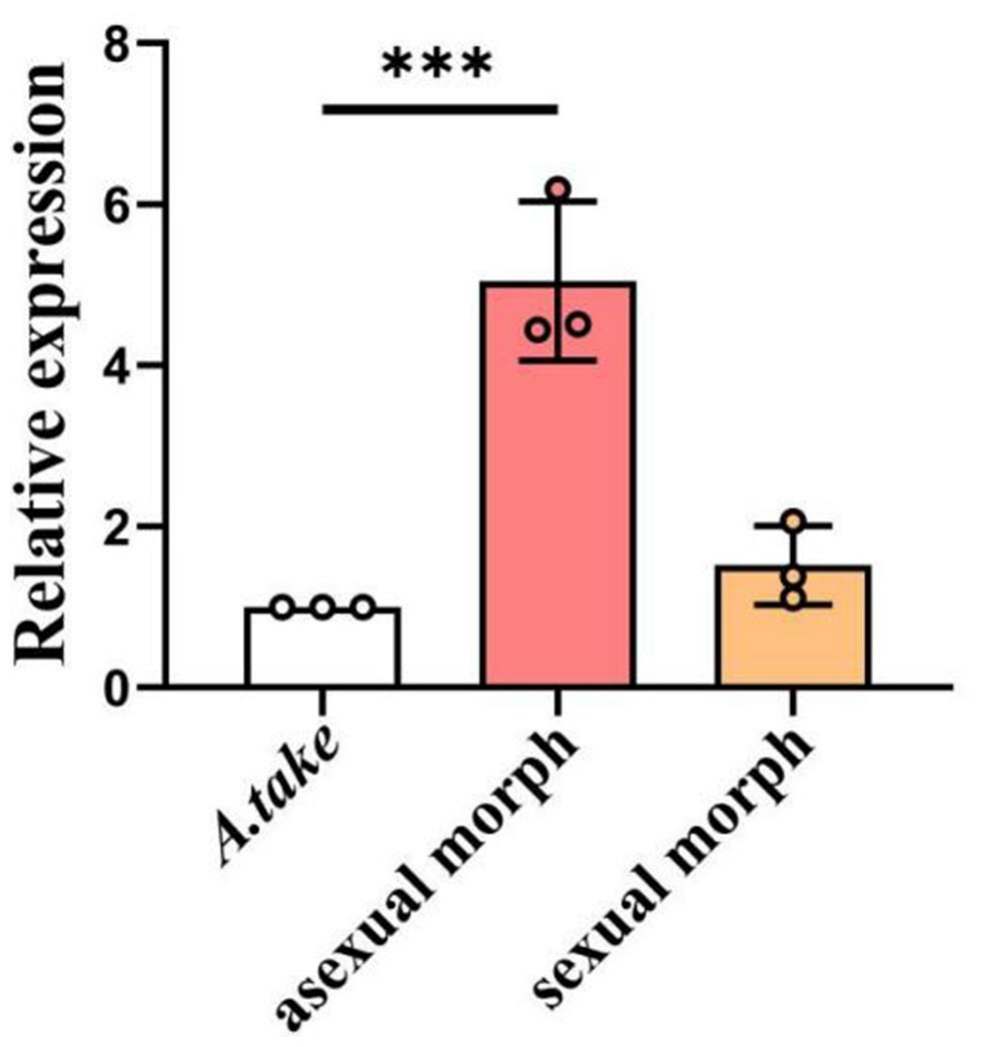
Relative expression of *miaA* gene in asexual and sexual structures collected from bamboo WBD symptomatic tissues in relation to *Aciculosporium take* culture in SDA. Statistical analysis was conducted using ANOVA method. Error bars indicate standard deviation (SD). ***p < 0.001.

During the asexual morph stage of *A. take*, bamboo apical shoots exhibited continuous growth, with apical shoots ceasing growth during the subsequent sexual morph stage, at which point lateral shoots began to develop. To investigate the involvement of the *miaA* gene in the pathogenesis of bamboo WBD, RT-qPCR was used to assess *miaA* expression in conidiostromata and stromata collected from WBD bamboo buds. A 5-fold change in *miaA* expression was observed during the asexual morph stage of the *A. take*, with a subsequent decrease in expression (1.9 times) during the sexual morph formation; nonetheless, expression remained elevated compared to *A. take* cultured in SDA (Figure 7).

## 4 Discussion

### 4.1 A high-quality genome of *A. take* was assembled

*Aciculosporium take* is a highly destructive WBD pathogen that has affected over 60 bamboo species in East Asia since the last century (Mohanan, 2002). Limited studies on identification and occurrence have been conducted on this pathogen, hindering effective disease control due to incomplete genomic information. Studying the molecular mechanisms of pathogen genes is a cost-effective way to prevent and control disease, with recent advances in high-throughput sequencing technologies leading to significant progress. The increase in DNA sequencing capabilities using NGS and SMRT technology and recent genome research on various species have opened up new opportunities to explore disease mechanisms at the molecular level.

In this study, the high-quality, chromosome-level genome assembly of *A. take* CCTCC-M2023413 was obtained using PacBio and Illumina sequencing platforms and Hi-C technology. The *A. take* genome size was determined to be 58.81 Mb with 7,105 protein-coding genes at a coverage of 109X. Hi-C mapping was used to assign 21 contigs to 7 chromosomes. BUSCO analysis showed 99.7% genome completeness in *A. take*. This percentage is higher than that of *Colletotrichum graminicola* (97.3%) (Becerra et al., 2023), *Phakopsora pachyrhizi* (90.19%) (Gupta et al., 2023), *Stagonospora tainanensis* (99.18%) (Xu et al., 2022) and *Fusarium oxysporum* f. sp. *albedinis* (98%) (Khayi et al., 2023). The high percentage of BUSCO close to 100% indicates a high-quality genome assembly for *A. take*.

Repetitive sequences, including TE, make up 54.32% of the *A. take* genome. Repetitive sequences are a source of mutations, gene polymorphisms, and sequence-specific DNA-binding proteins required to regulate gene expression (Alseekh et al., 2020). Despite having fewer TE than plants, fungi still rely on them for genome function (Depotter et al., 2022). *Botrytis cinerea* delivers regulatory trans-species small RNAs derived from LTRs into plant cells to suppress host gene expression, while LTRs expression in the pathogen further suppresses plant defense-related genes during infection (Porquier et al., 2021). In our study, we discovered that TE had the highest percentage of LTR components, as well as LINEs and DNA transposons. The most common type of LTRs was Copia retrotransposons, which is consistent with the fact that Copia-like retrotransposons form a large and ubiquitous class in the genome (Vukich et al., 2009). TEs were found in areas with high gene densities and many pathogenic-related factors. Changes in virulence in plant pathogenic fungi are linked to variations in repetitive insertion sequences. High-frequency TE can be used as DNA markers to distinguish different virulent strains, similar to Pot2 rep-PCR analysis in rice blast fungus.

### 4.2 *A. take* pathogenic factors are rich and diversity

In studying the pathogenic mechanism of *A. take*, various databases identified 237 carbohydrate active enzyme-related genes, 1,040 pathogen-host interaction genes, 315 virulence factor genes, and 70 effectors.

The GH, PL, and CE families of CAZYs, also known as cell wall-degrading enzymes (CWDEs) (Ospina-Giraldo et al., 2010), make up 50% of CAZYs in *A. take*. These enzymes are crucial for pathogenic fungi to break down plant tissues and obtain nutrients for growth and reproduction (Pavlov et al., 2018). GHs family enzymes, particularly GH3 *β*-glucosidases (EC 3.2.1.21), are important for degrading lignocellulose (Kubicek et al., 2014). VmGlu2, a GH3 enzyme in *Valsa mali*, is essential for the virulence of necrotizing pathogens (Huang et al., 2021). GH28 is involved in degrading pectin by removing ester groups, leading to the formation of pectin esters and methanol (Li et al., 2024). The GHs family is a significant part of *A. take*, with GH47 (9) and GH76 (8) being the most abundant genes. GH47, specifically, plays an important role in infecting grapevines (Onetto et al., 2022). Overall, lignocellulose degradation and polygalacturonase activity are key factors in *A. take* infection.

Studying membrane transport proteins can provide valuable information on substance transport and metabolism in microbes, aiding in understanding the pathogenic mechanisms and developing fungicide involved. The MFS family has the most members in *A. take*, which is important for maintaining fungal cell metabolism and growth, stress resistance, and drug resistance (Reddy et al., 2012). Further functional studies of MFS are warranted.

The pathogen-host interactions and virulence factors in plant pathogenic fungi are essential for genetic study. PHI-base provides phenotypic information on pathogenicity genes and host interactions. *A. take* has 1,040 PHI-associated genes (Figure 3C), which exceeded the number identified in the barley blast fungus *Pyrenophora teres* (594) (Bertazzoni et al., 2021).

### 4.3 Phytohormones synthesized by *A. take* are a key factor in inducing WBD

Recent reports suggest that phytohormone imbalance is a significant factor contributing to WBD (Tanaka, 2009; Teixeira et al., 2014). The study on the *A. take* genome identified a total of 227 genes related to the synthesis of three phytohormones: 172 genes for auxin synthesis, 53 genes for BR synthesis, and 2 genes for CK synthesis. Auxin induces plant cell wall relaxation, promoting cell elongation and longitudinal growth within the stem (Li et al., 2023), while BR plays a crucial role in regulating plant cell division, elongation, and differentiation (Planas-Riverola et al., 2019). Both auxin and BR promote plant cell enlargement and growth, while CK induces cell division and bud formation. In bamboo affected by WBD, it is hypothesized that *A. take* produces auxin and BR to stimulate growth and CK to inhibit apical meristematic tissue and promote lateral bud formation, resulting in the characteristic symptoms of WBD. Overall, the production of phytohormones acting on bamboo led to the typical WBD phenotype, but further experiments are needed to verify synthesis and pathogenesis mechanisms.

Analysis of PHIs results showed that CK synthesis key gene *miaA* (PHI:6266 and PHI:6267) was strongly linked to pathogenicity. Mutant phenotypes of auxin synthesis-related genes OPT (PHI:2976) and iaaH (PHI:4171) reduced virulence. Knockout of the gene in *Claviceps purpurea* strain Δ*CptRNA-ipt* resulted in reduced virulence, and the double knockout mutant lacking CK production was non-pathogenic (Hinsch et al., 2016). Genes related to CK and Auxin synthesis in *A. take* are linked to pathogenicity, consistent with WBD hormonal imbalance causing disease (Tanaka et al., 2003b).

The study of comparative genome found that all 13 WBD causal agents share the gene *miaA*, and *miaA* of *A. take* upregulated expression during the bamboo WBD pathogenesis, presuming that *cis*-zeatin is the main cause of WBD. To show *cis*-zeatin synthesis conservation in various WBD, a gene family analysis of miaA proteins in 13 pathogens was conducted. Gene structure is important for gene evolution. MiaA proteins in the same group have similar motifs and domains, indicating high conservation within the miaA family, which is crucial for understanding gene evolution and potential functional roles. Only two (*A. take* and *F. mangiferae*) of the 13 miaA protein sequences had the zinc-binding zf-U1 superfamily domain, which is unique to eukaryotes. Zinc finger motifs are important in host-pathogen interactions (Chen et al., 2021), highlighting the role of miaA in WBD pathogenesis. However, the current expression and bioinformatics analysis of *miaA* are insufficient to confirm its significant role, necessitating detailed functional studies for targeted fungicide development.

### 4.4 There are some common pathogenic factors among the causal agents of WBD

Comparative genomics analysis of 13 pathogens associated with WBD identified common pathogenic factors. *A. take* exhibited the closest genetic relationship to *F. mangiferae*, displaying significant chromosomal similarities, followed by *M. perniciosa*. Given the limited research on A. take, insights can be gleaned from studies on *F. mangiferae* (Cohen et al., 2017) and *M. perniciosa* (Barbosa et al., 2018). The remaining 10 species are phytoplasmas, distinct from fungi in terms of genome and characteristics, yet all 13 pathogens share 77 core orthogroups. The core orthogroups may play a role in causing WBD and further research is needed to understand the mechanisms.

Enrichment analysis by eggNOG and KEGG revealed that genes in these orthogroups are mainly involved in growth, metabolism, and genetic processing. The eggNOG annotation results revealed a predominance of genes in category J (translation, ribosomal structure, and biogenesis), while the KEGG annotation results indicated a higher number of genes in the Aminoacyl-tRNA biosynthesis pathway. Additionally, all species exhibit the presence of miaA, which catalyzes the conversion of adenosine 37 in tRNA to 6-isopentenyladenosine, a crucial step in the biosynthesis of *cis*-zeatin (Leung et al., 1997). The pathways for aminoacyl-tRNA biosynthesis and Zeatin biosynthesis both utilize tRNA as a substrate. We speculate whether gene expression in protein translation may be inhibited during pathogen infection, allowing more tRNA to be used for synthesizing *cis*-zeatin by miaA. Confirming this hypothesis could provide new insights for preventing and controlling WBD, but further gene expression data and metabolite testing are needed for confirmation.

All 13 WBD pathogens in category U have the signal recognition particle subunit SRP54 (K03106), which is involved in Quorum sensing and bacterial secretion system pathways in KEGG annotation. Quorum sensing allows bacteria to communicate and adjust gene expression based on cell density by releasing autoinducers (Li and Nair, 2012). In quorum sensing pathway of *Bacillus*, SRP54 regulates sporulation, virulence, and biofilm formation. *A. take* requires high concentrations of 10^6^ cfu/mL to grow and infect bamboo shoots, possibly related to SRP54. Meanwhile, the occurrence of WBD may be linked to specific signaling molecules produced by the pathogen.

The orthogroup genes shared by 13 pathogens were found to be enriched in pathways associated with longevity, such as the HIF-1 signaling pathway, FoxO signaling pathway, and Longevity regulating pathway. These genes encode proteins including pyruvate dehydrogenase E1 component (PHB, K00162), heat shock 70 kDa protein (HSPA, K03283), and superoxide dismutase (SOD, K04564). PHD in HIF-1 pathway regulates TCA cycle and oxygen homeostasis, while forkhead box O (FOXO) family regulate oxidative stress resistance and longevity genes. Dietary restriction and genetic down-regulation of nutrient-sensing pathways can increase a healthy lifespan (Green et al., 2022). Our hypothesis posits that the PHD enzyme creates a nutrient-deprived environment by inhibiting TCA cycle metabolism, activating the FoxO signaling pathway, and modulating the expression of SOD and HSPA in the longevity-regulating pathway, ultimately leading to an extension of lifespan. Following infection of bamboo buds by *A. take*, it typically takes 2–3 years for symptoms to manifest and for the fungus to begin producing spores. Additionally, the fungus remains dormant for 8 months out of the year, during which time infection branches cease to grow. It is hypothesized that pathogenic organisms can establish an interactive homeostasis with their host by extending itself lifespan, thereby preventing the premature death of the host resulting from ongoing growth and nutrient consumption. This potential survival and pathogenic mechanism may be common among arbuscular pathogens.

Analysis of the genome of *A. take* suggests that this fungal pathogen could induce disease in the host through the action of cell wall-degrading enzymes, fungal virulence factors, and modulation of plant hormone levels in conjunction with the host. The comparative genomic analyses produced significant findings, highlighting the essential function of the key zeatin synthesis gene, miaA, in WBD. Future research, should investigate if eukaryotic and prokaryotic pathogens use these similar strategies to cause WBD, aiding in broad-spectrum control methods.

## 5 Conclusion

We completed the assembly and annotation of a high-quality chromosome-scale reference genome of the pathogenic fungus *Aciculosporium take* CCTCC-M2023413 causing bamboo witches’ broom disease (WBD), integrating with PacBio sequencing, Illumina sequencing, and Hi-C technology. Numerous pathogenicity-related genes were identified, predominantly overlapping with repeat-rich regions of genome. Interestingly, *A. take* possesses the potential to synthesize phytohormones, comprehending auxin, brassinosteroid, and *cis*-zeatin. Comparative genomic analysis revealed that the *miaA* gene, key for *cis*-zeatin biosynthesis, is present in 13 pathogens causing plant WBD and is significantly induced in *A. take* during witches’ broom formation. This suggests that cytokinin secreted by *A. take* maybe a key factor in the pathogenesis of bamboo WBD, potentially providing a novel target for controlling this disease.

## Data Availability

The assembled sequence data reported in this study have been deposited in the Genome Sequence Archive (Genomics, Proteomics & Bioinformatics 2021) at the National Genomics Data Center (Nucleic Acids Res 2022), China National Center for Bioinformation, Beijing Institute of Genomics, and the Chinese Academy of Sciences (https://ngdc.cncb.ac.cn/gsub/), (Accession No.: CRA013419, publicly available data: 11 Nov. 2025).

## Footnotes

^1^ https://github.com/pacificbiosciences/unanimity

^2^ https://busco-archive.ezlab.org/v2/

^3^ http://xfam.org/

^4^ https://web.expasy.org/docs/swiss-prot_guideline.html

^5^ https://ftp.ncbi.nlm.nih.gov/blast/db/FASTA/nr.gz

^6^ http://geneontology.org/page/go-database

^7^ http://www.genome.jp/kegg/

^8^ http://www.ebi.ac.uk/interpro/

^9^ https://www.ebi.ac.uk/uniprot/

^10^ http://eggnog-mapper.embl.de/

^11^ http://pfam-legacy.xfam.org/

^12^ https://www.ncbi.nlm.nih.gov/cdd/

^13^ https://www.ncbi.nlm.nih.gov/genome/

^14^ http://meme-suite.org/
