# Supplementary figures and images for: Genomic insights into bamboo witches’ broom disease: pathogenicity and phytohormone biosynthesis in Aciculosporium take
Source: Front Microbiol. 2024 Nov 8;15:1432979. doi: 10.3389/fmicb.2024.1432979 (PMC11590067; doi:10.3389/fmicb.2024.1432979)

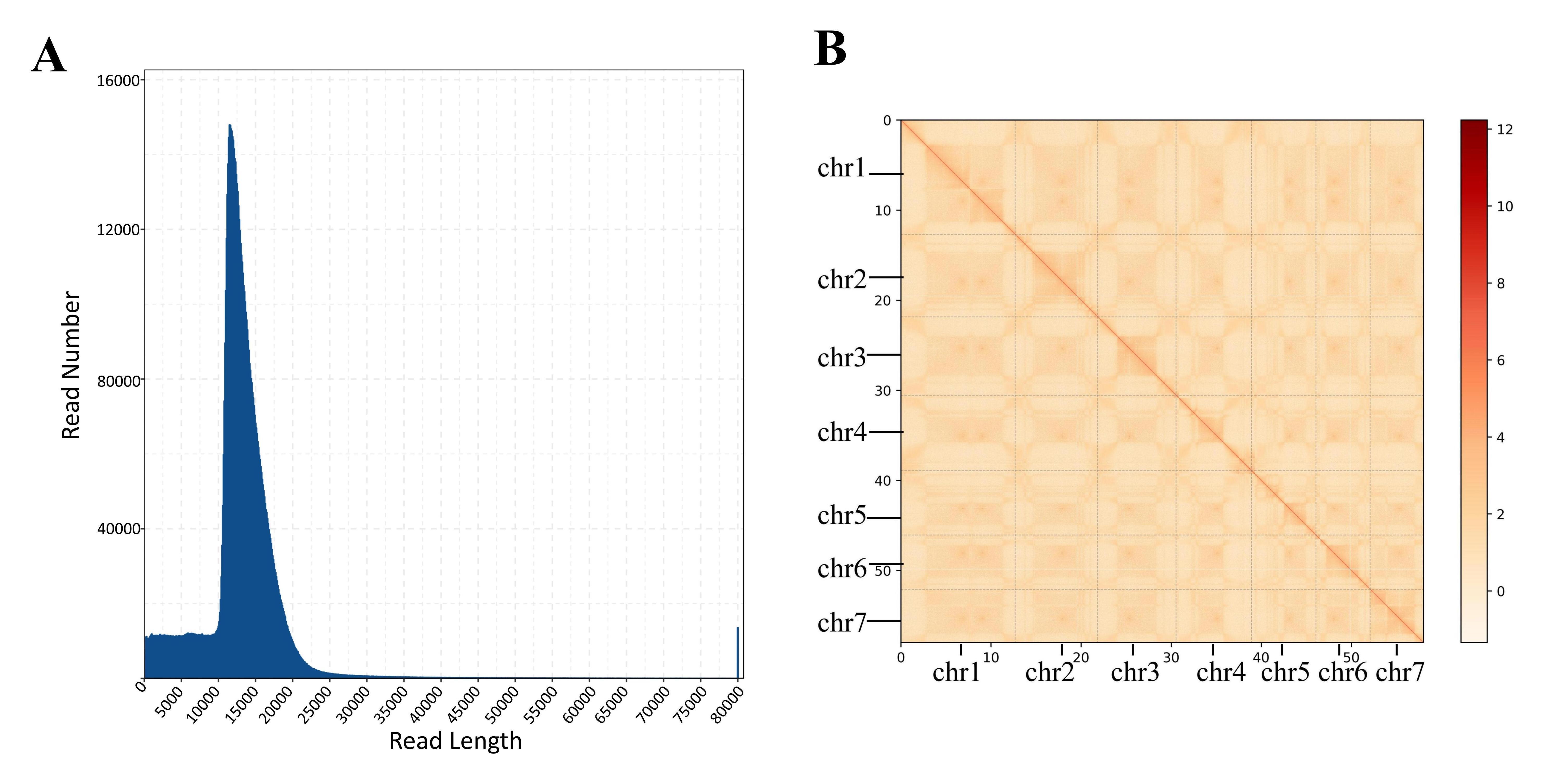

Supplement: Supplementary file 2 [file Image_1.JPEG]

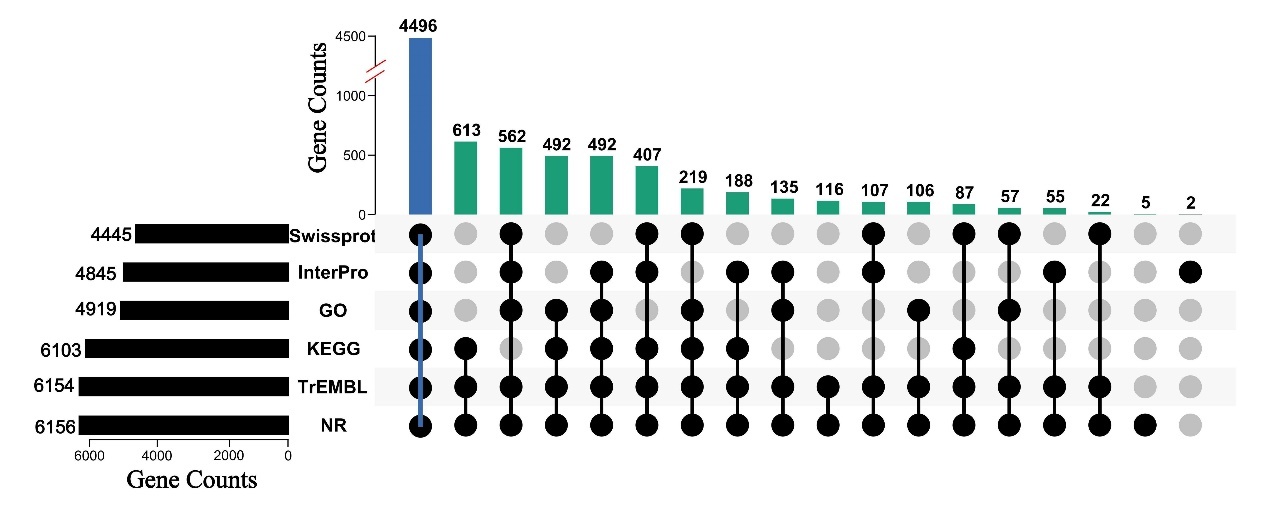

Supplement: Supplementary file 3 [file Image_2.JPEG]
